# Genotype-phenotype correlation in Japanese patients with familial Mediterranean fever: differences in genotype and clinical features between Japanese and Mediterranean populations

**DOI:** 10.1186/s13075-014-0439-7

**Published:** 2014-09-27

**Authors:** Dai Kishida, Akinori Nakamura, Masahide Yazaki, Ayako Tsuchiya-Suzuki, Masayuki Matsuda, Shu-ichi Ikeda

**Affiliations:** Department of Medicine (Neurology and Rheumatology), Shinshu University School of Medicine, 3-1-1 Asahi, Matsumoto, 390-8621 Japan; Intractable Disease Care Center, Shinshu University Hospital, 3-1-1 Asahi, Matsumoto, 390-8621 Japan; Department of Biological Sciences for Intractable Neurological Diseases, Institute for Biomedical Sciences, Shinshu University, 3-1-1 Asahi, Matsumoto, 390-8621 Japan

## Abstract

**Introduction:**

Familial Mediterranean fever (FMF) is a hereditary autoinflammatory disease characterized by recurrent self-limiting fever and serositis that mainly affects Mediterranean populations. Many patients with FMF have been reported in Japan due to increasing recognition of this condition and the availability of genetic analysis for the gene responsible, *MEFV*. The present study was performed to elucidate the clinical characteristics of Japanese FMF patients and to examine the precise genotype-phenotype correlation in a large cohort of Japanese FMF patients.

**Methods:**

We analyzed the *MEFV* genotypes and clinical manifestations in 116 patients clinically diagnosed as having FMF and with at least one mutation.

**Results:**

The most frequent mutation in Japanese patients was E148Q (40.2%), followed by M694I (21.0%), L110P (18.8%), P369S (5.4%), and R408Q (5.4%). In contrast, common mutations seen in Mediterranean patients, such as M694V, V726A, and M680I, were not detected in this population. The clinical features with M694I were associated with more severe clinical course compared to those seen with E148Q. P369S/R408Q showed variable phenotypes with regard to both clinical manifestations and severity. Patients with M694I showed a very favorable response to colchicine therapy, while those with P369S and R408Q did not.

**Conclusions:**

Clinical features and efficacy of treatment in Japanese FMF patients vary widely according to the specific *MEFV* gene mutation, and therefore genetic analysis should be performed for diagnosis in cases of Japanese FMF.

**Electronic supplementary material:**

The online version of this article (doi:10.1186/s13075-014-0439-7) contains supplementary material, which is available to authorized users.

## Introduction

Familial Mediterranean fever (FMF) is an autosomal recessive disorder characterized by recurrent, self-limiting episodes of fever accompanied by peritonitis, pleuritis, synovitis, and erysipelas-like skin lesions [1]. The patients having severe FMF attacks sometimes develop lethal renal amyloid A (AA) amyloidosis. Typical FMF attacks usually last for approximately 3 days, and they vary in frequency from once a week to several times per year. Oral colchicine therapy is quite effective for preventing attacks and the development of reactive AA amyloidosis. The gene responsible, *MEFV*, maps to the short arm of chromosome 16 and encodes a protein of 781 amino acids called pyrin [2,3]. Though the mechanism of pyrin action is still debated, it is considered to be a negative regulator of inflammation [4].

FMF affects more than 100,000 people around the world, and is particularly prevalent in the Mediterranean basin, occurring most commonly in Turks, Arabs, Armenians, and Jews. Five mutations, M694V, V726A, M680I, M694I (in exon 10), and E148Q (in exon 2), account for 74% of FMF mutations [5]. However, increasing numbers of cases have been reported in different ethnic groups and countries [6]. In Japan, cases of FMF with *MEFV* mutations were first reported in 2002 [7], and large numbers of FMF patients have been reported since even in Japan [8-13]. In addition, we described the clinical manifestations of FMF in 80 Japanese patients [14]. This study indicated that the severity of FMF is milder in Japanese than Mediterranean patients, with a lower frequency of AA amyloidosis, and the mutations are also different between Japanese and Mediterranean patients. However, FMF is still thought of as an uncommon disease in Japan, and the detailed clinical features associated with each type of *MEFV* mutation in Japanese patients have not been elucidated. To date, a number of clinical diagnostic criteria have been used to make a diagnosis of FMF [15-18]. However, it is possible that some Japanese patients would not meet these diagnostic criteria as they are based on the symptoms seen in Mediterranean patients. Therefore, many patients may remain undiagnosed and a comprehensive diagnosis, including genetic analysis, may be necessary especially in countries outside the Mediterranean area, such as Japan. The present study was performed to examine the precise relationships between each type of *MEFV* mutation and clinical manifestations in a large cohort of Japanese FMF patients. Moreover, we reconsidered the validity of the existing diagnostic criteria based on the genotype-phenotype correlation seen in Japanese FMF.

## Materials and methods

### Patients

*MEFV* mutation analyses were conducted on genomic DNA obtained from blood samples in patients with suspected FMF between September 2003 and December 2012. All patients provided informed consent prior to the genetic study for DNA testing of the *MEFV* gene and publication of their individual details under anonymity. A total of 216 patients from various hospitals in Japan were referred to our department for *MEFV* gene analyses. Clinical diagnosis of FMF was made according to the Tel-Hashomer criteria [15]. The major criteria are: 1) recurrent febrile episodes accompanied by peritonitis, synovitis, or pleuritis; 2) amyloidosis of the AA-type without predisposing disease; and 3) favorable response to colchicine treatment. Minor criteria were: 1) recurrent febrile episodes; 2) erysipelas-like erythema; and 3) FMF in a first-degree relative. Patients were divided into two groups based on the confidence of diagnosis: definitive FMF (two major or one major and two minor) and probable FMF (one major and one minor). A definitive or probable diagnosis could not be made in some cases despite recurrent febrile or painful episodes. To examine the genotype-phenotype correlations of Japanese FMF patients, we included a third group called suspected FMF (one major or one minor), as described previously [19]. In 216 patients, 76 were clinically evaluated by FMF experts in our hospital. Other 140 patients were evaluated by physician contributors throughout Japan using our original clinical survey sheet for FMF. Based on the clinical information, we have carefully assessed and determined the groups (definitive, probable, and suspected) in each patient. All the genetic analyses were conducted only in our hospital. Of the 216 patients, 147 carried certain *MEFV* mutation(s). Among these, 14 subjects were excluded on the basis of being asymptomatic family members of FMF patients. Moreover 17 patients were excluded due to the presence of different diseases: Behçet’s disease (n =5), inflammatory bowel disease (n =2), and sarcoidosis, mixed connective-tissue disease, adult-onset Still’s disease, granulomatosis with polyangiitis, pseudogout, malignant lymphoma, infectious spondylitis, intercostal neuralgia, mental disorder, and temperature instability (n =1) (Additional file 1). Consequently, we studied the clinical pictures and types of *MEFV* mutations of the remaining 116 patients. Among them, 39 patients who were diagnosed at our University and presented in our previous report [14] were included in this analysis. On the other hand, 69 patients had no mutation in the *MEFV* gene. Of 69 patients, 4 patients were definitive, 1 was probable, 39 were suspected, and 25 were not eligible according to the Tel-Hashomer criteria. This study was carried out in accordance with Institutional Review Board approval (No. 314) in the Shinshu University School of Medicine, Nagano, Japan.

### DNA testing of the *MEFV* gene

DNA analysis of the *MEFV* gene was performed in all 216 patients with suspected FMF. The five hotspot regions (exons 1, 2, 3, 5, and 10) for *MEFV* mutations were analyzed by PCR [20]. Exon 2 was amplified in two overlapping PCR fragments, designated as exon 2a and exon 2b. Amplified PCR products were analyzed by direct sequencing (DNA Analyzer 3730xl; Applied Biosystems, Foster City, CA, USA). Primers for PCR and sequence analysis were as follows: Exon 1 : 5'-TCC TAC CAG AAG CCA GAC AG-3′; Exon 1R: 5′-TTC CTG AAC TAA AGT CAT CT-3′; Exon 2aF: 5′-GCA TCT GGT TGT CCT TCC AGA ATA TTC C-3′; Exon 2aR: 5′-CTT TCC CGA GGG CAG GTA CA-3′; Exon 2bF: 5′-CAG GCC GAG GTC CGG CTG CG-3′; Exon 2bR: 5′-CTT TCT CTG CAG CCG ATA TAA AGT AGG-3′; Exon 3F: 5′-GAA CTC GCA CAT CTC AGG C-3′; Exon 3R: 5′-AAG GCC CAG TGT GTC CAA GTG C-3′; Exon 5F: 5′-TAT CGC CTC CTG CTC TGG AAT C-3′; Exon 5R: 5′-CAC TGT GGG TCA CCA AGA CCA AG-3′; Exon 10F: 5′-CCG CAA AGA TTT GAC AGC TG-3′; Exon 10R: 5′-TGT TGG GCA TTC AGT CAG GC-3′.

### Statistical analysis

We considered that to include more than 10 patients in each genotype was appropriate for statistical analysis. All data are expressed as the mean ± SD for continuous variables. Multiple differences between groups were compared by one-way analysis of variance (ANOVA) followed by Tukey’s post hoc test. Differences between categorical variables were analyzed using the chi-square test. In all analyses, *P* <0.05 was taken to indicate statistical significance. The statistical software, JSTAT [21], was used in the analyses.

## Results

### *MEFV* gene mutations

**Figure 1:**
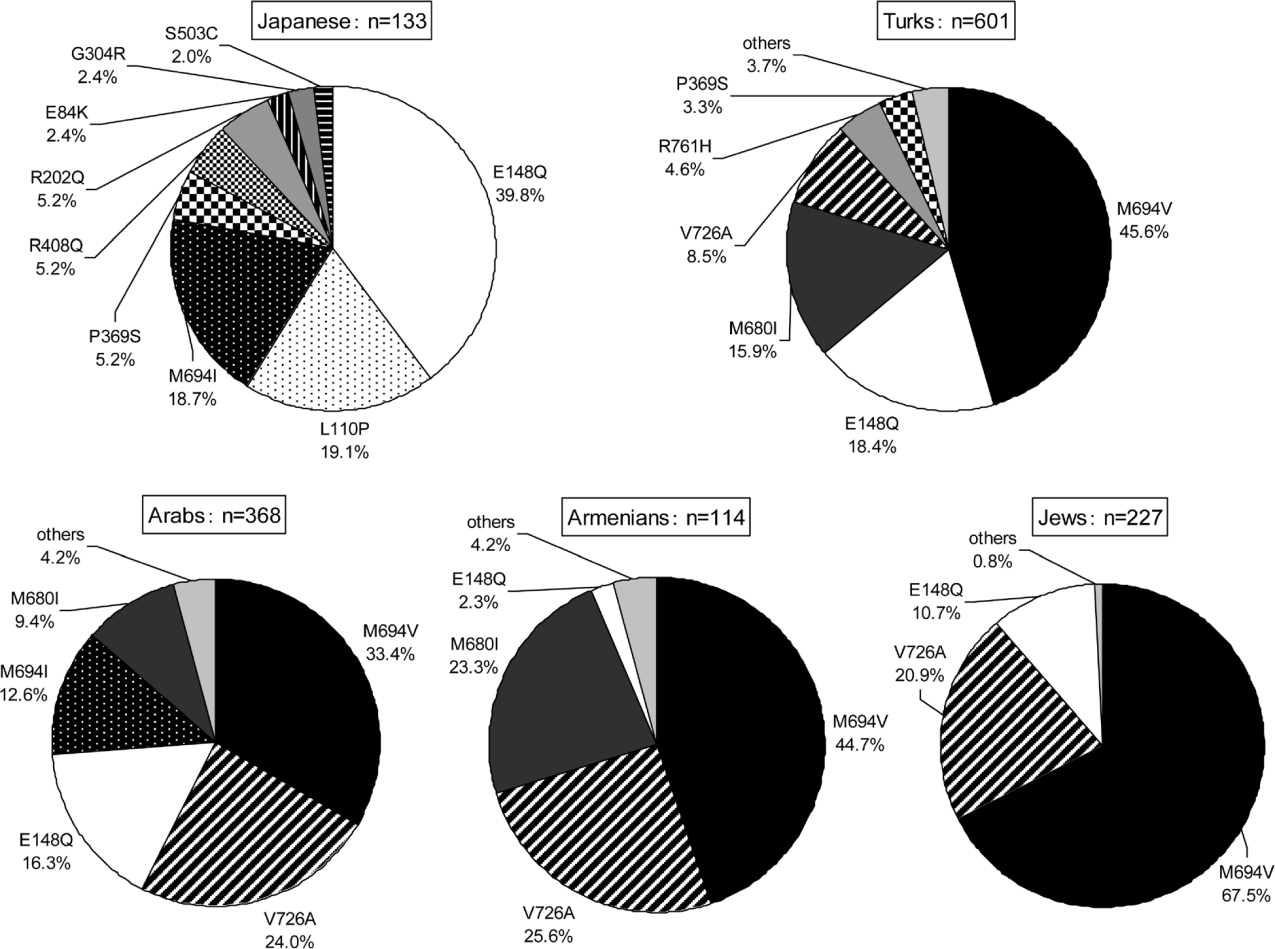
***MEFV*** **mutations in Japanese populations and Mediterranean populations** **[** 22 **-** 27 **].** n, number of patients with familial Mediterranean fever (FMF).

Among the 116 patients, 2 (1.7%) were homozygous, 67 (57.8%) were compound heterozygous, and 47 (40.5%) were heterozygous for *MEFV* mutations. Table 1 shows the distribution of *MEFV* mutations in this cohort. Complex alleles were observed in 32 (27.6%) patients. The detected mutations were E148Q (40.2%), M694I (21.0%), L110P (19.9%), P369S (5.4%), R408Q (5.4%), R202Q (4.5%), E84K (2.2%), S503C (1.9%), and G304R (0.9%). Repartition of the mutations in Japanese patients and comparison with findings in the four classically affected ethnic groups, that is, Turks, Arabs, Armenians, and Jews, are shown in Figure 1. Although M694V, V726A, and M680I are common mutations in the above four ethnic groups [22-27] these mutations were not detected in our study population.

**Table 1:** **Distribution of** ***MEFV*** **mutations**

**Homozygote**		**Compound heterozygotes**		**Heterozygote**	
**Mutation**	**Number**	**Mutation**	**Number**	**Mutation**	**Number**
M694I/M694I	2	E148Q/M694I	15	E148Q/wild-type	21
		L110P/E148Q/M694I	14	M694I/wild-type	13
		L110P/E148Q	13	R202Q/wild-type	8
		L110P-E148Q/E148Q	6	E84K/wild-type	3
		P369S/R408Q	4	S503C/wild-type	2
		L110P/E148Q/P369S/R408Q	4		
		E84K/L110P/E148Q	2		
		M694I/S503C	1		
		E148Q/S503C	1		
		L110P-E148Q/L110P-E148Q	1		
		L110P/E148Q/G304R	1		
		E148Q/P369S/R408Q	1		
		E148Q/E148Q-P369S-R408Q	1		
		E148Q/R202Q/P369S/R408Q	1		
		G304R/P369S/R408Q	1		
		E148Q/R202Q	1		
Total	2		67		47

### Clinical features

The male/female ratio in our study population was 51:65. The mean age at onset of symptoms was 23.7 ± 13.6 years, the mean frequency of attacks was 11.7 ± 11.3 per year, and the mean duration of attacks was 4.7 ± 7.7 days. The main clinical symptoms of the patients were as follows: high-grade fever with temperature >38°C was observed in 83.6% of the patients, peritonitis in 62.1%, pleuritis in 45.7%, arthritis in 41.4%, headache in 19.8%, myalgia in 11.2%, rash or erysipelas-like erythema in 6.8%, and amyloidosis in 1.7% of patients. Among the 116 patients, 70 (60.3%) were treated with colchicine and 64 (91.4%) had complete response (asymptomatic) or good response (occasional attacks). The average dosage of colchicine was 0.8 mg/day (0.25 to 2.0 mg/day); 51.6% of the patients received <0.5 mg/day and 84.4% received <1.0 mg/day.

### Genotype-phenotype correlation

**Figure 2:**
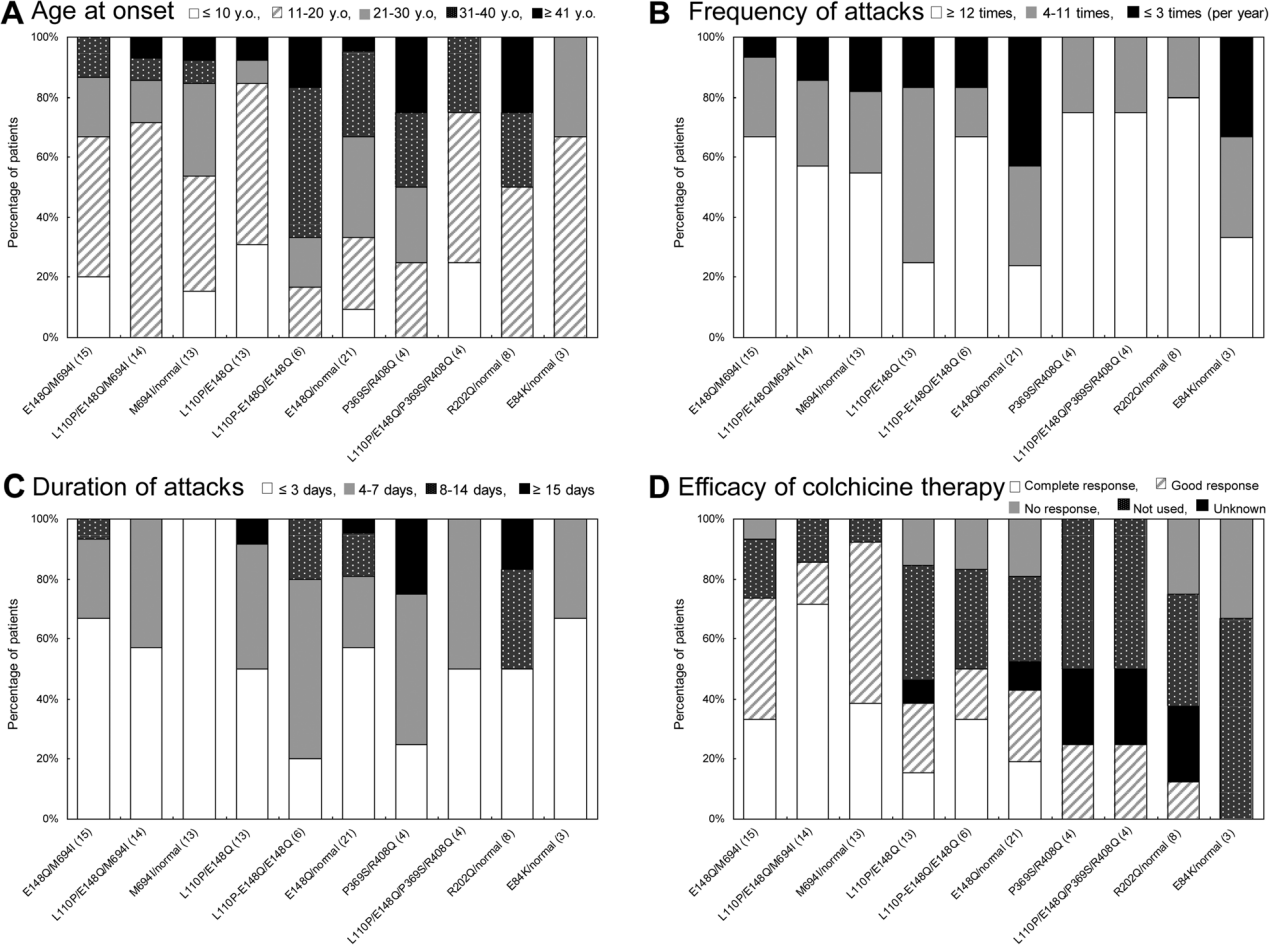
**Genotype-phenotype correlation of 116 Japanese familial Mediterranean fever (FMF) patients. (A)** Age at onset of each genotype in years (y.o.). **(B)** Frequency of attacks in each genotype (times per year). **(C)** Duration of attacks in each genotype (days). **(D)** Efficacy of colchicine therapy in each genotype. Complete response means asymptomatic, Good response means occasional attacks.

The genotype-phenotype correlation of all patients was evaluated in more than three patients with same mutation (Table 2), and in one or two patients with the same mutation (Additional file 2). We next examined the features of attacks according to age at onset, frequency, and duration of attacks. In the majority of patients carrying the M694I mutation (E148Q/M694I, L110P/E148Q/M694I, or M694I/wild-type), the age at onset was before 30 years, which was younger than for the other genotypes (Figure 2A). A portion of the patients who were not carrying M694I mutation had an age at onset >30 years and the disease developed in a few patients after age 40 years. The frequency of attacks in each genotype is shown in Figure 2B. More than 40% of patients carrying E148Q/wild-type experienced up to three attacks per year. Figure 2C shows the durations of attacks. Typically, the attacks in FMF patients resolve within 3 days [18], but the attacks lasted over 4 days in a number of patients. While attacks in all patients with M694I/wild-type improved within 3 days, 50.9% of patients not carrying this mutation had duration over 4 days.

**Table 2:** **Genotype-phenotype correlations in more than three patients with the same mutation**

	**Patients,** **number**	**Age at onset, years**		**Frequency of attacks per year**	**Duration of attacks (days)**	**Fever ≥38°C**	**Peritonitis**	**Pleuritis**	**Arthritis**	**Efficacy of colchicine**
		**Mean (± SD)**	**Range**	**Median (min, max)**	**Median (min, max)**	**%**	**%**	**%**	**%**	**%**
E148Q/M694I	15	18.8 ± 7.8	9 to 34	12 (2.5, 54)	2.5 (0.75, 8.5)	93.3	66.7	66.7	26.7	100 (n = 11)
L110P/E148Q/M694I	14	20.4 ± 8.7	13 to 46	12 (2, 30)	3 (2, 7)	85.7	85.7	78.6	50	100 (n = 12)
M694I/wild-type	13	20.2 ± 11.4	3 to 42	9 (3, 48)	2.5 (0.08, 3)	92.3	92.3	61.5	30.8	100 (n = 12)
L110P/E148Q	13	15.2 ± 11.6	2 to 48	5.5 (1.5, 18)	3.3 (1, 17.5)	84.6	69.2	0	38.5	83.3 (n = 6)
L110P-E148Q/E148Q	6	35.5 ± 15.1	18 to 65	12 (3, 12)	5 (0.75, 8.5)	50	66.7	50	16.7	100 (n = 3)
E148Q/wild-type	21	26.5 ± 12.1	7 to 61	4 (1, 24)	2.5 (1, 75)	81	52.4	42.3	47.6	81.8 (n = 11)
P369S/R408Q	4	35.5 ± 16.7	14 to 60	24 (6, 48)	5.8 (0.2, 19)	75	25	25	50	50 (n = 2)
L110P/E148Q/ P369S/R408Q	4	18.3 ± 8.9	6 to 31	18 (5, 24)	4 (2.5, 7)	100	25	50	25	50 (n = 2)
R202Q/wild-type	8	32.4 ± 19.4	11 to 68	15 (4, 48)	5.8 (1, 14)	87.5	50	25	75	33.3 (n = 3)
E84K/wild-type	3	20.7 ± 6.2	14 to 29	4 (2, 12)	*3* (1.5, 3.5)	100	0	33.3	33.3	NE

SD, standard deviation, NE, not examined.

**Figure 3:**
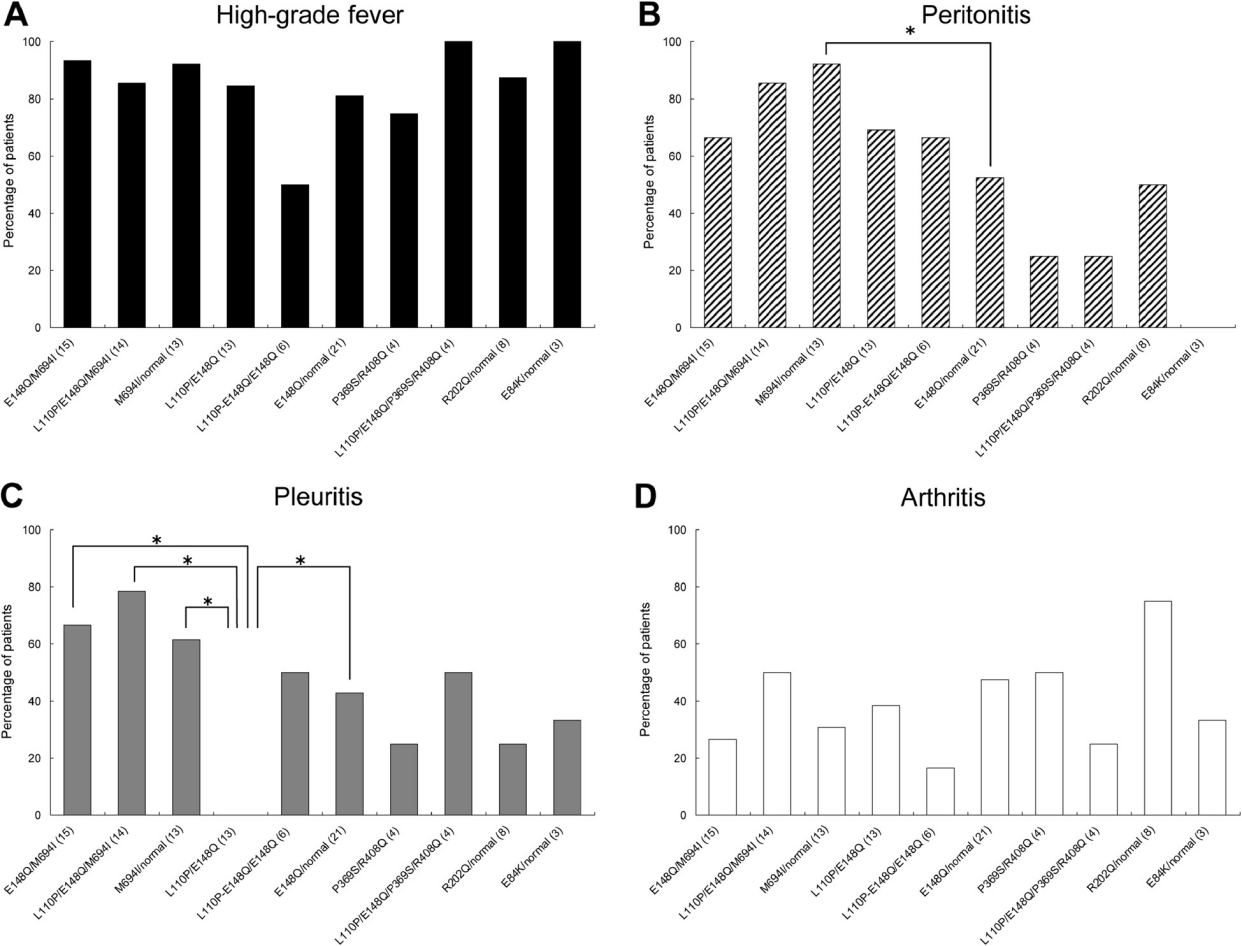
**Frequency of symptoms in each genotype.** High-grade fever (febrile episodes) **(A)**, peritonitis **(B)**, pleuritis **(C)**, and arthritis **(D)**. More than 10 patients in each genotype was used for the statistical analysis. *P <0.05.

We also analyzed the frequency of each symptom (fever, peritonitis, pleuritis, and arthritis). While high-grade fever (febrile episode; Figure 3A) was the most frequently observed symptom in each genotype group, 50% of the patients carrying L110P-E148Q/E148Q were afebrile or had low-grade fever. Peritonitis was observed in 92.3% of patients carrying M694I/wild-type, which was significantly higher than the patients carrying E148Q/wild-type (Figure 3B). Pleuritis was seen in over 60% of patients carrying the M694I mutation, but in under than 50% of those without this mutation (Figure 3C). Arthritis was observed in 75% of patients with R202Q/wild-type (Figure 3D). This symptom was only seen in fewer than 50% of patients with other genotypes.

Next, we evaluated the efficacy of colchicine therapy in each genotype group (Table 2, Figure 2D). Patients carrying the M694I mutation had an efficacy rate of 100% (complete response or good response). Colchicine therapy had good efficacy in patients with the L110P and/or E148Q mutation (L110P/E148Q, L110P-E148Q/E148Q, and E148Q/wild-type), although its rate of use was approximately 50%. In contrast, this treatment regimen had insufficient efficacy in patients with P369S/R408Q or R202Q/wild-type.

One patient with E148Q/M694I received a TNF-α receptor antagonist infliximab and low-dose methotrexate therapy because of colchicine intolerance for adverse events [28]. Further, some patients with the *MEFV* gene mutations were refractory to colchicine and had atypical symptoms of FMF. To these patients, certain biological drugs such as TNF-α and IL-1 receptor antagonists might be effective.

**Figure 4:**
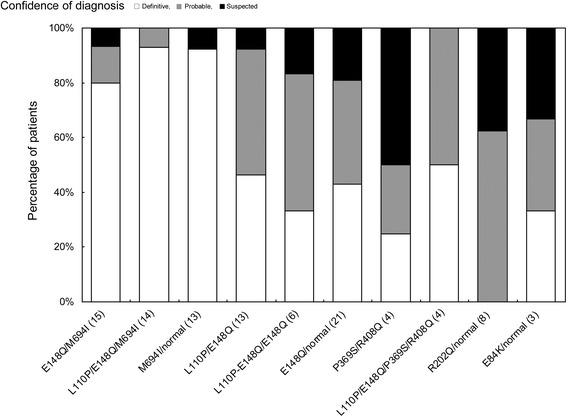
**Confidence of diagnosis based on the Tel-Hashomer criteria in each genotype.**

Finally, we examined the diagnostic rate using the Tel-Hashomer criteria in our patients with *MEFV* mutations (Figure 4). According to these criteria, 88.1% of patients carrying the M694I mutation matched the definitive diagnosis, while this was true for only 64.4% of patients without M694I as a probable or suspected diagnosis. In particular, large numbers of patients in the P369S/R408Q, R202Q/wild-type, and E84K/wild-type groups matched the criteria for a suspected diagnosis (Figure 4).

## Discussion

In this study, we examined the frequency of *MEFV* mutation, clinical manifestations, and the genotype-phenotype correlations in 116 Japanese patients with FMF. The clinical features of the Japanese patients were different from those seen in Mediterranean populations. Compared to Mediterranean patients, Japanese patients had late onset of symptoms, relatively low frequency of peritonitis, rarity of reactive AA amyloidosis, and low dosage of colchicine [14]. The results of the present study suggest that the differences in mutations between Japanese and Mediterranean populations are associated with differences in genotype. Over 280 sequence variants in the *MEFV* gene have been recorded in the Infevers database [29], the most common of which are M694V, M694I, V726A, M680I, and E148Q [5,19,30]. These five mutations account for approximately 90% of sequence variants in classically affected ethnic groups (Turks, Arabs, Armenians, and Jews) (Figure 1). However, only 61.2% of Japanese FMF patients in the present study had E148Q and/or M694I, and none of our patients had M694V, V726A, or M680I. Especially, M694V, which is related to the most severe clinical course [19,30], has not been detected in Japanese patients. On the other hand, the largest number of Japanese patients with the E148Q mutation had a mild clinical course [5]. It has been suggested that FMF, along with Behçet’s disease, may have been transmitted to Japan via travel along the Silk Road [6]. However, this hypothesis cannot explain the differences in mutation types between Japan and Mediterranean regions and the low prevalence of FMF, compared to Behçet’s disease, which is a common disease in Japan. As an island nation, Japan has a number of intrinsic disease features and Japanese patients have specific clinical presentations and mutation types.

As shown in Tables 1 and 2, the majority of Japanese FMF patients had some combination of M694I, E148Q, L110P, P369S/R408Q, and R202Q mutations. The presence of M694I in exon 10 is thought to be important for diagnosis and treatment. Patients with this mutation show a typical and severe clinical course, with early onset, high frequency and short duration of attacks, and high percentages of fever and serositis. However, their therapeutic response to colchicine is very good. Hence, identification of the M694I mutation is a useful diagnostic foundation for Japanese patients with suspected FMF.

E148Q has been considered to be a polymorphism because its allele frequency is high in healthy controls [31]. In Japan, it has been reported that allele frequency of E148Q, L110P, and M694I are 0.26, 0.039, and 0.0, respectively [14]. Nevertheless, several studies have indicated that most homozygous or compound heterozygous patients associated with other *MEFV* mutations are symptomatic and their clinical course is relatively mild [5,32]. In the present study, the clinical manifestations of patients with E148Q were later onset, lower frequency, and slightly longer duration of attacks, compared to patients with M694I. Moreover, some patients heterozygous only for E148Q presented with typical FMF manifestations. Generally, it is difficult to make a definite diagnosis in patients heterozygous for only E148Q. In such cases, a comprehensive diagnosis including not only the clinical course but also the efficacy of colchicine may be required.

The L110P mutation was always compound heterozygous with E148Q mutation, and there were no patients heterozygous for L110P alone. This mutation was identified commonly in our study and several cases of FMF with this mutation have been reported in Japan [33,34]. Although L110P may be a frequent mutation, especially in Japanese patients, there were no marked differences in the clinical picture between L110P/E148Q and E148Q alone.

In addition, it may be necessary to pay special attention to the diagnosis in patients with P369S/R408Q mutations in exon 3. P369S and R408Q are always identified in *cis* [35], as observed in our study. In the present study, 12 patients with P369S/R408Q (alone or with additional mutations) were identified, and their clinical features were characterized by a high frequency of attacks, variable phenotype, and incomplete efficacy of colchicine therapy. The mean frequency of attacks in these 12 patients was 22.5 times per year. Their clinical symptoms were highly variable: four were variable in frequency or duration of attacks, two experienced frequent abdominal attacks without high fever and elevation of serum C-reactive protein (CRP) level, and other findings. Eight patients received colchicine therapy, but four had only a partial response, and the others showed no response. Ryan *et al*. report that patients with P369S/R408S mutations have clinical diversity, atypical clinical presentation, and relative lack of response to colchicine treatment [35]. This report is comparable to the phenotype in our patients with P369S/R408S. Therefore, it will be necessary to establish other more effective therapies, including biological treatment, for such cases.

As indicated in the present study, there were differences in clinical characteristics between the various *MEFV* mutations. As the majority of patients with M694I have typical FMF symptoms, identification of this mutation can lead to a definite diagnosis. When other mutations, such as E148Q and/or L110P, P369S/R408Q, R202Q, E84K, S503C, or G304R are identified, a comprehensive diagnosis including the efficacy of colchicine treatment may be required. Therefore, it is important to perform *MEFV* gene analysis for diagnosis of patients with suspected FMF (recurrent febrile episodes and/or serositis, and exclude diagnosis of other diseases). After treatment with colchicine, it is important to examine the diagnostic validity. The possibility of FMF cannot be excluded even in the absence of detectable *MEFV* mutations, but a careful colchicine trial is required.

## Conclusions

The results of the present study indicated that the clinical characteristics and *MEFV* genotypes of Japanese FMF patients are different from those seen in Mediterranean populations. Our clinical data established a spectrum of *MEFV* mutations among Japanese patients. As clinical features and efficacy of treatment vary widely according to the mutation(s) present, comprehensive diagnosis including clinical data and genetic analysis is needed for Japanese FMF patients.

## Additional files

Additional file 1:
**The 17 patients excluded due to diagnosis of other diseases.**
Additional file 2:
**Genotype-phenotype correlations in selected patients.**

## Abbreviations

AA: amyloid A
FMF: familial Mediterranean fever
IL: interleukin
MEFV: Mediterranean fever
NE: not examined
PCR: polymerase chain reaction
TNF: tumor necrosis factor
