# Supplementary material for: Genotype-phenotype correlation in Japanese patients with familial Mediterranean fever: differences in genotype and clinical features between Japanese and Mediterranean populations
Source: Arthritis Res Ther. 2014 Sep 27;16(5):439. doi: 10.1186/s13075-014-0439-7 (PMC4201677; doi:10.1186/s13075-014-0439-7)
Supplement: Additional file 1: — The 17 patients excluded due to diagnosis of other diseases. [file 13075_2014_439_MOESM1_ESM.doc]

Additional file 1 Excluded 17 patients for diagnosed other different disease
