# Supplementary material for: Genotype-phenotype correlation in Japanese patients with familial Mediterranean fever: differences in genotype and clinical features between Japanese and Mediterranean populations
Source: Arthritis Res Ther. 2014 Sep 27;16(5):439. doi: 10.1186/s13075-014-0439-7 (PMC4201677; doi:10.1186/s13075-014-0439-7)
Supplement: Additional file 2: — Genotype-phenotype correlations in selected patients. [file 13075_2014_439_MOESM2_ESM.doc]

Additional file 2 Genotype-phenotype correlations in selected patients

NE, not examined. +, positive for symptom, ‐, negative for symptom.
